# Investigating school absenteeism and refusal among Australian children and adolescents using Apriori association rule mining

**DOI:** 10.1038/s41598-024-51230-4

**Published:** 2024-01-22

**Authors:** Umme Marzia Haque, Enamul Kabir, Rasheda Khanam

**Affiliations:** 1 School of Mathematics, Physics and Computing, University of Southern Queensland, Toowoomba, Australia; 2 School of Business, University of Southern Queensland, Toowoomba, Australia

**Keywords:** Psychology, Mathematics and computing

## Abstract

Identifying and determining the multitude of reasons behind school absences of students is often challenging. This study aims to uncover the hidden reasons for school absence in children and adolescents. The analysis is conducted on a national survey that includes 2967 Australian children and adolescents aged 11–17. The Apriori association rule generator of machine learning techniques and binary logistic regression are used to identify the significant predictors of school absences. Out of 2484, 83.7% (n = 2079) aged (11–17) years children and adolescents have missed school for various reasons, 42.28% (n = 879) are (11–15) years old, 24.52% (n = 609) and 16.9% (n = 420) are 16- and 17-years old adolescents respectively. A considerable proportion of adolescents, specifically 16.4% (n = 407) and 23.4% (n = 486) of 16 and 17 years old, respectively, have selected ‘refused to say’ as their reason for not attending school. It also highlights the negative outcomes associated with undisclosed reasons for school absence, such as bullying, excessive internet/gaming, reduced family involvement, suicide attempts, and existential hopelessness. The findings of the national survey underscore the importance of addressing these undisclosed reasons for school absence to improve the overall well-being and educational outcomes of children and adolescents.

## Introduction

The phenomenon of school refusal and absenteeism is a significant concern that can have detrimental effects on mental and physical well-being of an individual. Research studies have shown potential consequences of school refusal behavior, including the development of mental disorders, substance abuse, aggressive behavior, and self-harm^1–8^. These consequences are commonly associated with anxiety, fear, depression, somatic symptoms, tiredness, social disengagement, sleep disturbances, self-consciousness, mood disorders, and disruptive behaviour problems^9–13^.

In recent times, several research studies have investigated the factors associated with school refusal and absenteeism. To identify the key factors influencing this behaviour, literature from areas such as psychology, social/criminal justice, and education has been reviewed^1^. The results of these studies have shown a significant association between personality dimensions and school refusal behaviour among Spanish students aged 8–11^14^. Based on previous research studies on school absenteeism and dropout, criteria for inclusion and exclusion have been formulated to identify the risk factors^15^.

In addition, a multi-tiered system of support framework (MTSS) has been used to identify various aspects that align well with school absenteeism and its problems^16^. In order to develop a school absenteeism system that can classify worries, a text classification method with machine learning (ML) has been used to tag posts on an online application system through discussions with students^17^. Additionally, ensemble, classification, and regression tree analysis have helped identify potential internalizing behaviour risk factors among youths at different levels of school absence severity^18^.

ML-based algorithms such as Random Forest (RF), Support Vector Machine (SVM), Boosted Regression, and Post-LASSO have been utilized by researchers to examine risk factors as potential early warning signs of school absence^19^. These algorithms have also been used to identify students with distinct risk indicators for not finishing high school on time^20^. Moreover, a study has focused specifically on clinic-referred children and adolescents aged 10–14 from primary and secondary schools in Melbourne, Australia, who were refusing to attend school and had at least one anxiety problem^21^.

Furthermore, a study has conducted on young people aged 10–17 who had been diagnosed or treated for school refusal behaviour between 1994 and 1998 at the Rivendell Unit in Sydney, Australia, found a high prevalence of mood and disruptive behaviour disorders^22^. Chi-square and Anova tests have been used to analyse the data in this study. Although numerous studies have been conducted on the topic of school refusal and absenteeism, the majority of them have been focused on Europe, Asia, the United States, and Canada, with only a few being have been carried out in Australia. This discrepancy in research has created methodological gaps in the existing evidence.

Many of the earlier studies have primarily concentrated on high school students, specifically 9th graders, making it difficult to obtain accurate statistics. Moreover, past research has relied on ML or statistical methodologies to identify specific behaviours associated with these issues, mainly for predictive modelling and classification. However, these methodologies do not investigate students’ behaviour and activities to determine the genuineness of their reasons for absences and the underlying factors contributing to this phenomenon.

Additionally, most studies have relied on clinical referrals or discussions, leading to a lack of research utilizing large, nationally representative datasets to examine absenteeism. In particular, there is a lack of research using association rule mining to investigate students’ behaviour and activities, which can provide valuable insights into the underlying reasons for absences. Furthermore, most studies have relied on information from clinical referrals or discussions when exploring the topic of absenteeism. Consequently, there is a lack of research utilizing large, nationally representative datasets to examine this problem, especially using association rule mining to investigate students’ behaviour and activities to determine the genuineness of their reasons for absences and the underlying factors contributing to this phenomenon.

Association rule mining is an effective technique for uncovering patterns and relationships in large datasets^23,24^. By identifying frequent itemset and association rules based on co-occurrence relationships, this method allows for the discovery of hidden patterns and associations that may not be apparent through other techniques. When it comes to school absences, association rule mining can help reveal interesting relationships between different factors contributing to absences and provide valuable insights into the underlying reasons behind them.

Despite the effectiveness of association rule mining in uncovering interesting associations or patterns in data, it has not been utilized in any previous studies. Hence, this study aims to employ association rule mining to identify the genuine reasons for school absences and pinpoint at what point it develops into school refusal. Given the absence of prior research employing a large dataset to examine this phenomenon, the present study aims to ascertain the underlying factors contributing to these behaviours by an analysis of data derived from young minds matter (YMM), a nationwide survey in Australia that focuses on mental health and overall well-being. Overall, this study investigates how association rule mining can be applied to discover the hidden information by analysing huge amount of data from YMM to create potentially meaningful patterns to extract the most relevant features related with school refusal and absenteeism to identify, in particular:

1. Which children refuse to attend school,
2. What are the reasons for their absence,
3. Most importantly, what are the underlying factors contributing to school absenteeism among children and adolescents, and at what point does it transition into school refusal, is there anything that parents, teachers, or school officials should be aware of?

To accomplish this, the study has been utilized the Apriori algorithm, a widely recognized machine learning algorithm for association rule mining^25–31^. This algorithm has been widely employed in various fields such as hypothesis testing, numerical analysis, and large-scale data processing^32,33^. Given the lack of prior research utilizing a large dataset to examine this phenomenon, this study aims to uncover the hidden information and create meaningful patterns to extract the most relevant features related to school refusal and absenteeism.

## Results

**Figure 1:**
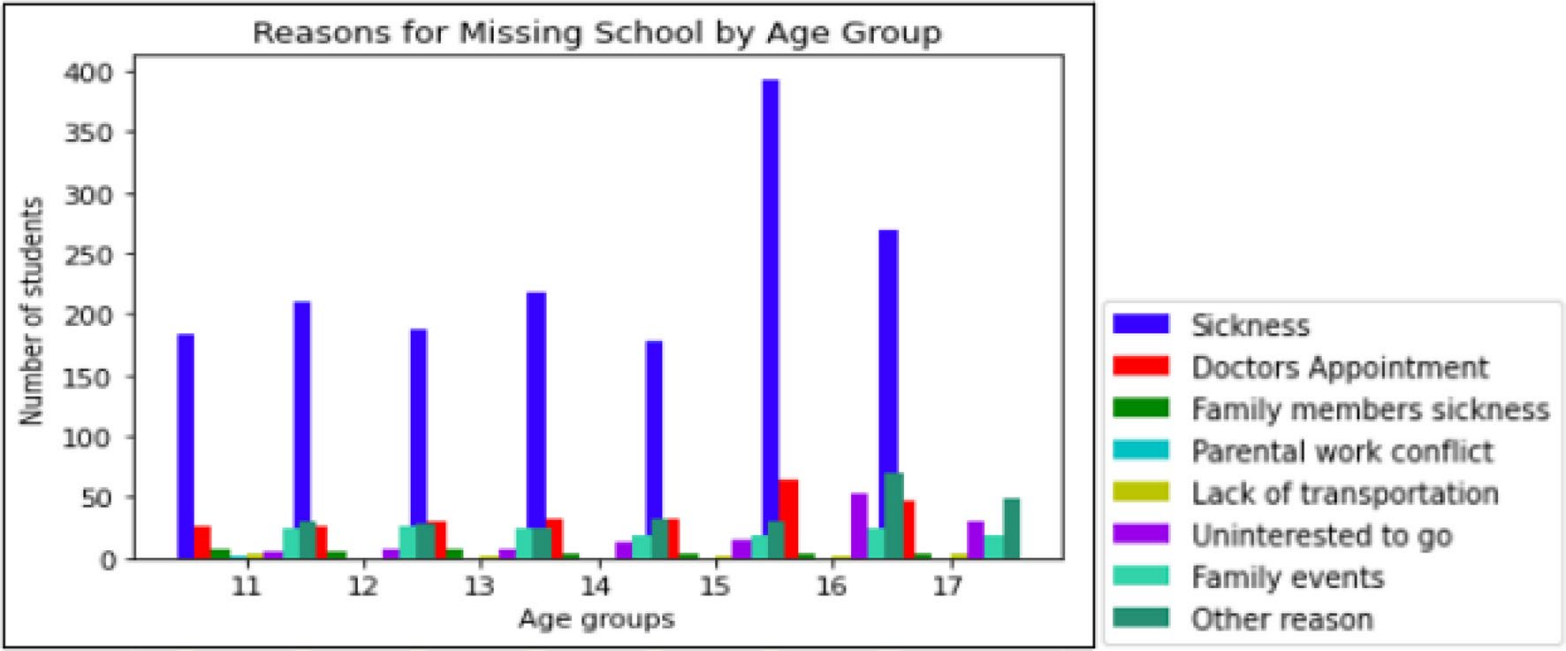
Students who missed schools for various reasons by age.

The analysis has begun with the question, ‘What was the primary reason for missing school?’. The YMM dataset provides data on 2484 children who did not attend school. Out of these, 1639 children were sick, 256 had medical appointments, 33 had family members who were sick, 1 child faced parental work conflict, 10 lacked transportations, 128 did not want to go to school, 154 had family events, and 263 had other reasons. Figure 1 categorizes these children by age and the aforementioned reasons. This information is crucial in understanding why students are absent from school.

Figure 1 displays that the students who missed school are aged between 11 and 17 years. Notably, a significant percentage of students (12%, 41%, and 23%) in the 15–17 age group express a lack of motivation to attend school. To gain deeper insights into this resistance, analysing the data using the Apriori association rule mining technique would be valuable. This technique helps identify patterns and relationships among the reasons for missing school, shedding light on the underlying factors contributing to their lack of attendance. By employing this technique, patterns and relationships can be uncovered, aiding in our understanding of why students are not attending school.

### Apriori algorithm analysis

The Apriori algorithm, a data mining technique, has been utilized in this analysis to discover associations or relationships among items in the dataset. The algorithm generates frequent item sets, which are sets of items that frequently appear together in transactions. These frequent item sets are then used to generate association rules that describe the relationships between items.

When conducting association rule mining, antecedents and consequents are determined based on statistically significant relationships between variables in the dataset. The specific antecedents and consequents can vary depending on the research question and the analysis being conducted.

In this analysis, the Apriori algorithm has been applied to the YMM dataset to identify associations between factors related to students’ lack of interest in attending school. The algorithm selects rules with higher lift and conviction values, indicating the strength and reliability of the associations. The associated factors of disinterest in going to school lead to interesting sub-issues related to research objectives, outlined in Table 1.

**Table 1:** Associated factors related to the disinterest in going to school.

Consequent	Antecedent	Support	Confidence	Lift	Conviction
Not interested in going to school	Felt life was not worth living	0.03	0.34	2.98	1.35
	Easily distracted	0.03	0.35	2.99	1.36
**Associated factors of the feeling life was not worth living**					
Felt life was not worth living	Bullied by others	0.02	0.31	5.10	1.36
	Easily distracted	0.02	0.30	4.93	1.34
	Attempt suicide	0.02	0.29	4.90	1.33
	Spend less time with family	0.03	0.30	4.86	1.33
**Associated factors of easily distracted**					
Easily distracted	Worry a lot	0.05	0.65	1.39	1.52
	Restless	0.05	0.65	1.39	1.52
	Angry	0.05	0.65	1.39	1.52
	Go without eating/sleeping because of internet or electronic game	0.04	0.63	1.36	1.45
**Associated factors of bullied by others**					
Bullied by others	Spend less time with family	0.03	0.55	3.16	1.84
	Restless	0.03	0.55	3.16	1.84
	Easily distracted	0.03	0.55	3.06	1.77
**Associated factors of attempting suicide**					
Attempted suicide	Worry a lot	0.03	0.47	14.25	1.83
	Felt life was not worth living	0.02	0.47	14.13	1.81
**Associated factors of spending less time with family**					
Spend less time with family	Worry a lot	0.02	0.44	2.38	1.46
	Easily distracted	0.02	0.44	2.38	1.46
	Restless	0.02	0.42	2.26	1.41
	Do you feel bothered when you can’t be on the internet/ play electronic games?	0.02	0.42	2.26	1.41
	Felt life was not worth living	0.02	0.41	2.19	1.38
**Associated factors of worry a lot**					
Worry a lot	Restless	0.04	0.50	1.29	1.23
	Bullied by others	0.04	0.49	1.26	1.19
	Easily distracted	0.04	0.49	1.26	1.19
**Associated factors of restless**					
Restless	Easily distracted	0.03	0.55	2.66	1.77
**Associated factors of angry**					
Angry	Easily distracted	0.03	0.39	2.98	1.36
	Felt life was not worth living	0.03	0.39	2.98	1.36
**Associated factors of go without eating/sleeping because of internet or electronic game**					
Go without eating/sleeping because of internet or electronic game	Do you feel bothered when you can’t be on the internet/ play electronic games?	0.03	0.55	1.07	1.01
**Associated factors of feeling bothered without browsing internet/playing electronic games**					
Do you feel bothered when you can’t be on the internet/play electronic games?	Go without eating/sleeping because of internet or electronic game	0.03	0.37	2.30	1.34
	Felt life was not worth living	0.03	0.37	2.30	1.34
	Spend less time with family	0.03	0.32	2.00	1.24
	Restless	0.03	0.32	2.00	1.24
	Worry a lot	0.03	0.31	1.91	1.22

Table 1 presents the associated factors related to disinterest in going to school. The Apriori algorithm is applied to the first consequent as ‘Not interested in going to school’. It uncovers two strong associated antecedents as ‘Felt life was not worth living’ (lift: 2.98, conviction: 1.35) and ‘Easily distracted’ (lift: 2.99, conviction: 1.36).

However, it is important to note that the identification of antecedents and consequents does not imply a one-way relationship. Rather, it suggests that the presence of the antecedents increases the likelihood of observing the consequents. Additionally, these antecedents can themselves be influenced by other factors, which is why this analysis continues to explore associations with these identified antecedents.

In the second phase of analysis, the resulted antecedents from the first consequent have been set as consequents to explore the other associated factors regarding these factors. In this analysis, significant links have been found between ‘Felt life was not worth living’ and ‘Bullied by others’ (lift: 5.10, conviction: 1.36), ‘Easily distracted’ (lift: 4.93, conviction: 1.34), ‘Attempt suicide’ (lift: 4.90, conviction: 1.33) and ‘Spend less time with family’ (lift: 4.86, conviction: 1.33). Furthermore, ‘Easily distracted’ is found to have four associated antecedents: ‘Worry a lot’ (lift: 1.39, conviction: 1.52), ‘Restless’ (lift: 1.39, conviction: 1.52), ‘Angry’ (lift: 1.39, conviction: 1.52), and ‘Go without eating/sleeping because of internet or electronic game’ (lift: 1.36, conviction: 1.45).

This analysis is continued to investigate the underlying causes of these associated factors whenever a strong association is discovered. For example, ‘Bullied by others’ is explored and found to have ‘Spend less time with family’ (lift: 3.16, conviction: 1.84) as a strongly associated antecedent.

Another consequent, ‘Spend less time with family’, is significantly linked to the antecedent ‘Do you feel bothered when you can’t be on the internet/play electronic games?’ (lift: 2.26, conviction: 1.41). This factor, in turn, is associated with ‘Go without eating/sleeping because of internet or electronic game’ (lift: 2.30, conviction: 1.34) and ‘Spend less time with family’ (lift: 2.00, conviction: 1.24). Association rules are built based on lift and conviction values greater than 1, indicating significant rules, even though the minimum confidence level is set at 34.8% in Table 1.

The identification of an item as both an antecedent and a consequent can occur when there are strong relationships between multiple variables in the dataset. This circular relationship can be a result of complex interactions among various factors influencing the behaviour under investigation.

**Figure 2:**
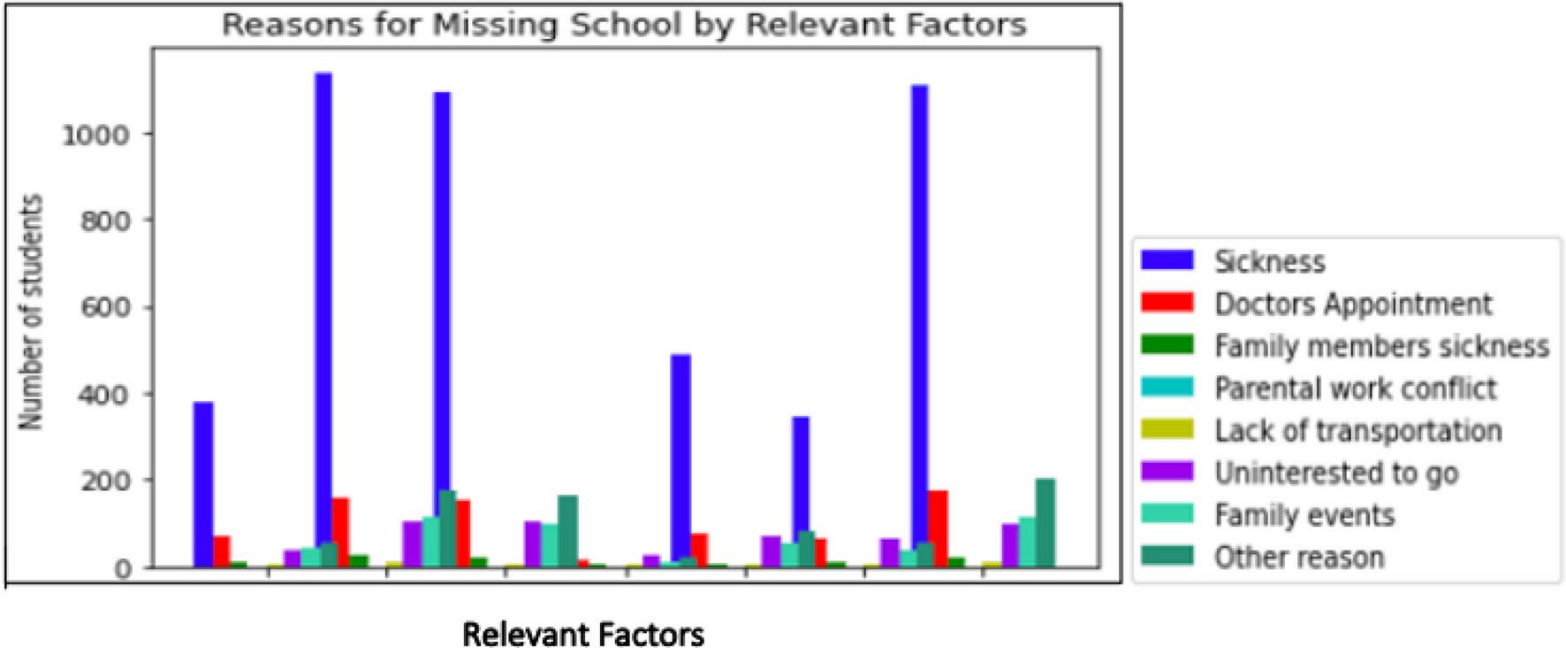
Students who miss school for various reasons influenced by associated factors.

Based on the values of lift and conviction from the resulted antecedents, the underlying causes of new antecedents such as being bullied by others, attempting suicide, spending less time with family, worrying a lot, feeling restless, feeling angry and feeling bothered when not on the internet/playing electronic games have been investigated. These factors are found to have a significant impact on children’s emotional and behavioural issues, as shown in Fig. 2.

Figure 2 indicates that a significant proportion of children and adolescents who have been absent due to illness also have encountered additional challenges or issues. Specifically, 23% (n = 377) of them have reported being victims of bullying, while 69.37% (n = 1137) have displayed a dependency on electronic games or excessive internet use. Additionally, 66.50% (n = 1090) of them, have showed a lack of prioritization when it comes to spending time with their families. This behaviour could potentially be attributed to their engagement in gaming or excessive internet use, or their reluctance to reveal their emotional state resulting from bullying experience. These reasons are also evident in other cases. For children and adolescents who missed school for a doctor’s appointment, 26.56% (n = 68) are bullied and 62.5% (n = 160) have reported developing dependencies on internet, playing electronic games, and 60.54% (n = 155) of them spend less time with their families. The percentages are 27.27% (n = 42), 74.68% (n = 115) and 64.29% (n = 99) for children who missed school for family events.

Although 263 children and adolescents have stated that they had other reasons for missing school, they did not specify whether bullying, internet addiction, electronic games, and spending less time with family are contributing factors for their school absence. The answer has been found in the summary shown in Fig. 2, which reveals that 20.53% (n = 54), 65.78% (n = 173), and 61.59% (n = 162) are affected by bullying, internet/electronic game addiction, and a lack of time spent with family members, respectively. These factors have been found to be significant reasons for school absences, as demonstrated by age in Table 2.

**Table 2:** Influence of school missing factors by age.

Reason	Age							
	11 n (%)	12 n (%)	13 n (%)	14 n (%)	15 n (%)	16 n (%)	17 n (%)	Total n (%)
Bullied	99 (35.48)	99 (32.78)	98 (34.63)	80 (25.47)	56 (20.29)	100 (16.42)	58 (13.81)	590 (23.75)
Bothered without internet/electronic games	199 (71.33)	215 (71.19)	201 (71.02)	232 (73.89)	192 (69.57)	424 (69.62)	257 (61.19)	1720 (69.24)
Less family time	141 (50.54)	164 (54.30)	189 (66.78)	220 (70.06)	208 (75.36)	431 (70.77)	282 (67.14)	1635 (65.82)
Attempt suicide	0 (0)	10 (3.31)	14 (4.94)	22 (7.01)	20 (7.25)	66 (10.84)	36 (8.57)	168 (6.76)
Skip eating/sleeping because of internet/electronic game	70 (25.09)	71 (23.51)	81 (28.62)	101 (32.17)	104 (37.68)	210 (34.48)	147 (35)	784 (31.56)
Felt life was not worth living	0 (0)	53 (17.54)	52 (18.37)	74 (23.57)	73 (26.45)	200 (32.84)	123 (29.29)	575 (23.15)
Restless	191 (68.46)	210 (69.54)	194 (68.55)	212 (67.52)	207 (75)	428 (70.28)	286 (68.09)	1728 (69.57)
Easily distracted	166 (59.49)	190 (62.91)	190 (67.14)	222 (70.70)	195 (70.65)	444 (72.91)	295 (70.24)	1702 (68.52)
Worry a lot	143 (51.25)	170 (56.29)	181 (63.96)	191 (60.83)	194 (70.29)	448 (73.56)	298 (70.95)	1625 (65.42)
Angry	137 (49.10)	145 (48.01)	124 (43.82)	155 (49.36)	138 (50)	302 (49.59)	183 (43.57)	1184 (47.67)

Table 2 highlights the significant prevalence of bullying incidents among children and adolescents who have experienced school absences. The highest percentage of bullying incidents is observed among 11-year-old children at 35.48% (n = 99). Additionally, a significant percentage of children experiencing school absences, ranging from 61 to 71%, develop addictions to internet usage or electronic gaming. Among 15-year-old adolescents, the highest percentage of 75.36% (n = 208) is observed, and they also report spending less time with their families.

Moreover, a significant proportion of these children (23.15%; n = 575) have expressed a belief that life is not worth living. They have also developed unhealthy habits such as skipping meals or lacking sufficient sleep. This issue is particularly prominent among the age group of 15 to 17 years old, with percentages of 37.68% (n = 104), 34.48% (n = 210), and 35% (n = 147) respectively.

To examine the association between school absences and various factors, the Apriori algorithm has been used. While this analysis has identified several potential factors related to school absences, it is important to note that association does not necessarily imply causation. To determine the best predicted factors and explain school absences among children, a multivariate approach, specifically binary logistic regression, has been employed.

### Multivariate analysis

After identifying the contributing factors for school absences by uncovering the underlying pattern of the variable, a determination has been made regarding their significance. In order to conduct a multivariate analysis, a binary logistic regression has been employed^34^. All potential factors identified through the Apriori algorithm analysis have been used as independent variables in the binary logistic regression. The coefficient and odds ratio have been examined with a 5% error rate to investigate the strength of these relationships.

It is worth noting that a few of these factors do not reach significance based on the conventional 95% confidence interval. In this analysis, the dependent variable is whether a child or adolescent missed school, represented by ‘1’ for absences and ‘0’ for attendance. The estimates, odds ratios (OR), and 95% confidence intervals (CI) can be found in Table 3.

**Table 3:** Binary logistic regression results for school absence across the various factors.

Reference category	Coefficient	OR	95% CI
Bullied	0.26	1.30	(0.98, 1.70)
Bothered without internet/electronic games	0.26	1.29	(1.06, 1.58)
Spent less family time	0.22	1.25	(0.98, 1.58)
Attempted suicide	0.51	1.66	(1.19, 2.31)
Skipped eating/sleeping because of internet/electronic game	0.25	1.29	(0.50, 3.31)
Felt life was not worth living	0.55	1.74	(1.19, 2.53)
Restless	0.05	1.05	(0.82, 1.34)
Easily distracted	0.07	1.07	(0.85, 1.37)
Worry a lot	0.06	1.06	(0.85, 1.33)
Angry	− 0.13	0.88	(0.65, 1.18)

Table 3 presents the outcomes of a binary logistic regression analysis, which investigates the relationships between school absenteeism and the various factors identified through the Apriori algorithm analysis. Based on the results presented in Table 3, it can be observed that children and adolescents who have developed dependencies on internet usage or electronic gaming are approximately 1.29 times more likely to be absent from school compared to their counterparts (OR: 1.29, 95% CI: 1.06, 1.58). Other significant factors associated with school absenteeism include suicide attempts and the belief that life is not worth living. Children who have attempted suicide and express feelings that life is not worth living are 1.66 times (OR: 1.66, 95% CI: 1.20, 2.31) and 1.74 times (OR: 1.74, 95% CI: 1.20, 2.52) more likely to miss school than their respective counterparts.

## Discussion

The children and adolescents have provided specific reasons for their school absences in this study. By using the Apriori algorithm on a large dataset from YMM, Australia’s recent nationally representative survey, this study has identified 10 associated factors out of 534 variables related to school absenteeism. Notably, bullying, addiction to internet/electronic games, spending less time with family, suicide attempts, and feelings of hopelessness have been found to be significant factors using association rule mining contributing to school absences among Australian children and adolescents. Some of these associated factors have been determined to be significant through the implementation of binary logistic regression analysis. The analysis reveals that while some of the significant factors from association rule mining do not reach statistical significance at the 5% level, they still provide meaningful insights into the relationships and patterns within the dataset. Association rule mining evaluates these associations based on strength and reliability measures such as lift and conviction.

It is worth noting that association rule mining can uncover other significant relationships and patterns in the dataset, even if they do not meet the strict criteria for statistical significance. The emphasis is placed on the strength and reliability of the connections between variables as indicated by lift and conviction values. Therefore, associations identified through association rule mining should still be considered meaningful and valuable, as they provide insights into the dataset, regardless of their statistical significance. Entirely, this research both confirms and expands on previous findings in this area^2,35–37^.

Previous studies have mainly focused on mental disorders^17,22,38^ and limited aspects of school functioning, such as teacher’s behaviour^39^, interaction^40–42^, safety^43–45^, while overlooking factors like bullying, internet/game addiction, lack of family time, and feelings of hopelessness. Interestingly, students have not consistently disclosed these factors as reasons for their absences. The use of association rule mining has uncovered hidden information, suggesting that students may develop disinterest or aversion towards school, eventually leading to school refusal.

Furthermore, this study has identified the age groups most impacted by bullying and internet/electronic game addiction, with the highest percentage observed among individuals aged 11 and 15, respectively. The study has also revealed a significant prevalence of suicidal ideation, skipping meals and sleep among students, particularly prominent among individuals aged 15–17. These findings demonstrate the potential of association rule mining to uncover hidden information and gain deeper insights into the reasons behind school absenteeism and school refusal.

Unlike previous studies that rely on existing literature or use a limited number of variables and participants, this research is based on a comprehensive Australian national dataset, including children and adolescents aged 11–17, capturing a crucial period in their academic development. The large and diverse sample enhances the applicability of the findings to a wider population.

The results highlight the importance of parents, teachers, and school authorities being aware of these significant factors contributing to school refusal or absenteeism, as they have a detrimental impact on students’ learning. It is observed that while other children attend school, these particular children express a desire to stay at home to engage in internet browsing or play electronic or online games. In accordance with existing research, the results of this study have shown that this reliance has negative consequences such as aggressive behaviour, social isolation, a loss of sense of reality, and health issues such as vision loss and hearing problems^46–48^. Additionally, attention should be given to the content these children access on the internet, particularly concerning issues like pornography, violence, terror, or gambling, as they can contribute to unethical thoughts and behaviours that are harmful to both the children and society^49^.

## Limitation of the study

There are a few limitations that need to be acknowledged in regard to the study. Firstly, it is important to note that the sample used in this study is limited to Australia. Therefore, the findings and conclusions may not be applicable to other countries or populations. However, it is worth mentioning that the study has analysed a comprehensive Australian national dataset, which included children and adolescents aged 11–17 years. The large sample size and diverse range of participants enhance the potential generalizability of the research findings. Additionally, it is important to recognize that the study relied on yes–no categorical variables. While this approach may not fully capture the complexities of the factors contributing to school absenteeism and the development of a school refusal attitude, it does provide a straightforward and clear method for examining the presence or absence of certain factors related to school absenteeism. This simplification aids the analysis process and can lead to actionable recommendations. Another limitation to consider is that the research excluded ‘Unknown’ categories, which could potentially result in the loss of valuable information and influence the findings and conclusions. Nevertheless, the outcomes of this model illustrate the effectiveness of the data building template in determining the factors associated with school refusal and absenteeism behaviours.

## Conclusion

Attending school is the only way for learning to expand the options and improve overall chances of success. Therefore, it is essential to identify the causes of school refusal and absenteeism behaviour in children and adolescents. In this study, Apriori has proven to be an efficient association rule generator for determining the associated factors of school refusal and absenteeism behaviour using YMM, a large dimensional dataset of children and adolescents’ mental health in Australia. Moreover, the results from the logistic regression model reveal that being bullied, bothered without internet/electronic games, suicide attempt, and feeling that life is not worth living are the most significant factors for missing school. Surprisingly, children and adolescents did not include these as reasons for school absence. Furthermore, Apriori identifies several other characteristics related to school refusal and absenteeism behaviour in children and adolescents, such as restlessness, being easily distracted and angry, worrying, although these are not statistically significant in logistic regression. The serious implications of school refusal and absenteeism on a student’s future prospects, including lower incomes, higher unemployment rates, and compromised health, make it imperative for parents, teachers, and school officials to understand the significance of these newly identified contributing factors. By taking these factors into account, school attendance can be prioritized as a fundamental concern.

## Materials and methods

**Figure 3:**
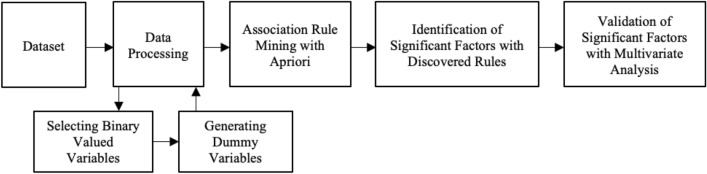
Functional pattern of the proposed method.

The phenomenon of school refusal and absenteeism among children and adolescents is a multifaceted problem that is influenced by various causes. In order to understand and address this behaviour, a comprehensive model has been developed to examine the underlying causes. The framework of the study analysis is shown in Fig. 3.

### Dataset

In this study, the factors responsible for school refusal and absenteeism of the children and adolescents have been detected using YMM, a nationwide cross-sectional Australian data organized by the University of Western Australia (UWA) for the Telethon Kids Institute. It is funded by the Australian Department of Health^50^. This dataset is available by submitting a request to the Australian Data Archive (ADA) at https://dataverse.ada.edu.au. The data collection process has received ethical approval from the Human Research Ethics Committees of AGDH and UWA, respectively^50,51^. YMM data has been collected using a multi-stage, area-based random sample technique. It has been designed to be representative of Australian families with children aged 4–17. If a family had more than one eligible child, the survey has been given to one of them at random. A total of 6310 parents/careers (55% of eligible households) of children and adolescents aged 4–17 voluntarily participated in the study.

### Data processing

#### Variable selection

This study focuses on the selection of categorical variables, specifically those with binary values of ‘Yes’ and ‘No’. Categories such as ‘Do not know’, ‘Refused’, ‘Missing’, ‘Not Available’, ‘Null’ are replaced with ‘Unknown’ value. Similarly, values such as ‘Yes—A lot’, ‘Yes—Minor’, ‘Yes—Minor difficulties’, ‘Yes—Severe difficulties’, ‘Yes—Sometimes’, ‘Fairly often’, ‘Very often’, and ‘True’ are replaced with ‘Yes’ to treat them as instances of experiencing difficulties. Categories like ‘Not at all’ and ‘Never’ are replaced with ‘No’ to capture the absence or lack of something. This grouping of similar responses into binary categories creates a more manageable dataset that can be easily interpreted by the model. The aim is to capture the underlying patterns and relationships between variables, rather than focusing on the specific values themselves.

While binary representation may not capture the nuances of the original responses, it is a trade-off made to simplify the analysis and enhance the model’s ability to generalize and make accurate predictions. This approach allows for the identification and understanding of significant patterns and trends, even if some detailed information about the original values is sacrificed.

Any category with more than 2000 ‘Unknown’ values is excluded from the analysis. Out of the remaining variables, 533 categorical variables with ‘Yes’/‘No’ have been selected. Additionally, 3 categorical variables (named ‘year of school’, ‘main reasons of missing school’ and ‘age’) with multiple values (where year of school and age are quantitative variables) have also been selected. In total, 536 variables have been selected from the original dataset, which initially comprised 680 variables. The column values have been converted to numeric values using the factorize() function to encode the string variables.

#### Dummy variable creation

Dealing with multiple values in the data input can pose challenges for the model’s ability to accurately comprehend and interpret the data. This can result in the model failing to recognize recurring patterns and treating them as separate entities, leading to inaccurate forecasts. To address this issue, it is recommended to use dummy variables, which effectively represent different categories, especially when dealing with numerous instances in the input characteristics. This approach enhances the model’s understanding and assimilation of the data, ultimately leading to more precise predictions. To simplify the process of uncovering associations between variables using the pandas.get_dummies() function, each variable in the dataset has been coded as either ‘Yes’ or ‘No’ with corresponding numerical values. Thus, a dummy variable has been created for each potential value, where 0 signifies ‘No’, and 1 signifies ‘Yes’.

#### Target variable

The variable pertaining to the question ‘What was the primary reason for missing school?’ has eight categories that explain the reasons for missing school. These categories include sickness, doctor’s appointments, family members’ sickness, conflicts with parental work, lack of transportation, lack of interest in attending school, family events, and other reasons. Dummy variables have been created for each of these categories. In order to analyse the causes of school absenteeism and refusal among Australian children and adolescents, the category of ‘lack of interest in attending school’ has been selected as the target variable.

### Methodology

The Python 3.7.3 sci-kit-learn package has been used to create a machine learning model using the association rule learning technique. Specifically, the Apriori algorithm, which is a well-known algorithm for association rule mining, has been applied to discover the variables that frequently occur together and contribute to certain behaviours in the YMM dataset.

#### Association rule mining

Association rule mining is a method used to uncover important patterns and associations in large datasets^23,52^. It involves identifying correlations between items, events, or variables and generating rules that capture these associations. The aim is to extract rules that express relationships between various items in the dataset, typically in the form of ‘if–then’ statements, where the antecedent (if-part) represents the presence of certain items or events, and the consequent (then-part) represents the occurrence of other items or events^53^. This feature of item association discovery, along with its ability to be applicable across different domains and its lack of prior assumptions, makes association rule mining an invaluable tool in data mining and analytics.

#### Apriori

The Apriori algorithm is widely recognized as the primary method for association rule mining and discovering new patterns of association^32,33,54^. In this study, the Apriori has been used to analyse patterns of student behaviour, specifically in identifying relationships between different reasons for missing school. By using the Apriori algorithm, frequently occurring combinations of absence reasons are identified, which provide insights into associations between variables related to school absenteeism and refusal attitude in the YMM dataset. The Apriori methods that have been followed in this study are as follows^55^:

- Itemset generation: Identification of frequently occurring variables, example: If X and Y are two variables, then (X, Y) is a representation of the list of all items which form the association rule
- Rule generation: Finding interesting patterns and trends between variables, example: (X → Y) is a representation of finding Y in all items which has X on it
- Apriori principle: Construction of all subsets of frequently occurring variables by diving them into two components such as antecedent and consequent
- Apriori algorithm: Cleaning the deductive rules and selecting the association rules based on interestingness measure such as support, confidence, lift and conviction
- Maximal frequent itemset: Identification of the frequently encountered variables such that none of the immediate variables are frequently encountered
- Closed frequent itemset: Identification of frequently occurring variables such that no other frequently occurring variables have the same support value

#### Performance measure

To evaluate the performance of the method, four metrics are calculated: support, confidence, lift, and conviction^56^:

Support:

Support indicates the frequency of an item appearing in the dataset. The support for the combination X and Y will be the following equation:

1 $${\text{Support}}({\text{X}} \to Y) = \frac{{\text{Transactions containing both X and Y}}}{{\text{Total Transactions}}}$$

Confidence:

Confidence measures the reliability of a rule. It is the conditional probability of the consequent (Y) given the antecedent (X) that can be measured with following equation:

2 $${\text{Confidence}}\;({\text{X}} \to Y) = \frac{{\text{Transactions containing both X and Y}}}{{\text{Transactions containing Y}}}$$

Lift:

Lift quantifies the strength of association between the components of a rule which is measured through the equation:

3 $${\text{Lift}}({\text{X}} \to Y) = \frac{{{\text{Support}}\left( {{\text{ X}} \to {\text{Y }}} \right)}}{{{\text{Support}}\left( {\text{X}} \right){\text{*Support}}\left( {\text{Y}} \right)}}$$

Conviction:

Conviction calculates the probability of one event occurring without another when they are dependent on each other, and this can be calculated using the following formula.

4 $${\text{Conviction}}\;({\text{X}} \to Y) = \frac{{1 - {\text{ Support}}\left( {\text{Y}} \right)}}{{1 - {\text{Confidence}}\left( {{\text{X}} \to {\text{Y}}} \right)}}$$

The Apriori algorithm uses these metrics to evaluate the strength and likelihood of association between the rule body and the rule head. Support refers to the proportion of transactions in the dataset that contain both the rule body and the rule head, lift measures the strength of association, and conviction measures the likelihood of the rule head occurring given that the rule body has already occurred. If support is less than 1 but lift and conviction are greater than 1, it suggests that although the rule occurs infrequently in the dataset, there is a strong association between the rule body and the rule head. High lift and conviction indicate that the occurrence of the rule body has a positive effect on the occurrence of the rule head, even if the overall support for the rule is low.

To ensure a high degree of accuracy and strong relationships between variables, a minimum support value of 3% (min_support = 0.03) has been set. This parameter is used by the Apriori method to reduce candidate rules by establishing a minimum lower bound for the support measure of the generated association rules.

It is important to note that association does not imply causality, despite the multiple connections between predictors of school absenteeism in children and adolescents uncovered through association rule mining. Therefore, a multivariate methodology has been employed to determine the optimal predictive variables and explain the phenomenon of school absenteeism. Estimates, odds ratios, and confidence intervals have been used to assess statistical significance of the findings.

## Data Availability

The authors declare that they do not have permission to share dataset. However, this dataset is available by submitting a request to the Australian Data Archive (ADA) at https://dataverse.ada.edu.au.

## References

[1] Kearney CA (2008). An interdisciplinary model of school absenteeism in youth to inform professional practice and public policy. Educ. Psychol. Rev..

[2] Kearney CA (2008). School absenteeism and school refusal behavior in youth: A contemporary review. Clin. Psychol. Rev..

[3] Kearney CA, Gonzálvez C, Graczyk PA, Fornander MJ (2019). Reconciling contemporary approaches to school attendance and school absenteeism: Toward promotion and nimble response, global policy review and implementation, and future adaptability (Part 1). Front. Psychol..

[4] Almeida MDCC, Aquino EM, Barros APD (2006). School trajectory and teenage pregnancy in three Brazilian state capitals. Cadernos de Saúde Pública.

[5] Chou L-C, Ho C-Y, Chen C-Y, Chen WJ (2006). Truancy and illicit drug use among adolescents surveyed via street outreach. Addict. Behav..

[6] Denny SJ, Clark T, Watson PD (2003). Comparison of health-risk behaviours among students in alternative high schools from New Zealand and the USA. J. Paediatr. Child Health.

[7] Guttmacher S, Weitzman BC, Kapadia F, Weinberg SL (2002). Classroom-based surveys of adolescent risk-taking behaviors: Reducing the bias of absenteeism. Am. J. Public Health.

[8] Henry KL, Huizinga DH (2007). Truancy’s effect on the onset of drug use among urban adolescents placed at risk. Journal of Adolescent Health.

[9] Egger HL, Costello JE, Angold A (2003). School refusal and psychiatric disorders: A community study. J. Am. Acad. Child Adolesc. Psychiatry.

[10] Gonzálvez C (2019). Relationship between school refusal behavior and social functioning: a cluster analysis approach. Eur. J. Educ. Psychol..

[11] Jones AM, West KB, Suveg C (2019). Anxiety in the school setting: a framework for evidence-based practice. Sch. Ment. Heal..

[12] Kearney CA, Albano AM (2004). The functional profiles of school refusal behavior: Diagnostic aspects. Behav. Modif..

[13] Maynard, B. R., Brendel, K. E., Bulanda, J. J., Thompson, A. M. & Pigott, T. D. Psychosocial interventions for school refusal behavior with primary and secondary school students: A Campbell systematic review and meta-analysis. *Society for Research on Educational Effectiveness* (2015).

[14] Martín M (2021). School refusal behavior profiles, optimism/pessimism, and personality traits in Spanish children. Educ. Sci..

[15] Gubbels J, van der Put CE, Assink M (2019). Risk factors for school absenteeism and dropout: a meta-analytic review. J. Youth Adolesc..

[16] Kearney CA, Graczyk PA (2020). A multidimensional, multi-tiered system of supports model to promote school attendance and address school absenteeism. Clin. Child Fam. Psychol. Rev..

[17] Ishikura, R., Takeda, M. & Iwashita, S. in *2020 Joint 11th International Conference on Soft Computing and Intelligent Systems and 21st International Symposium on Advanced Intelligent Systems (SCIS-ISIS)* 1–3 (IEEE).

[18] Fornander MJ, Kearney CA (2020). Internalizing symptoms as predictors of school absenteeism severity at multiple levels: Ensemble and classification and regression tree analysis. Front. Psychol..

[19] Chung JY, Lee S (2019). Dropout early warning systems for high school students using machine learning. Child. Youth Serv. Rev..

[20] Aguiar, E. *et al.* in *Proceedings of the Fifth International Conference on Learning Analytics and Knowledge* 93–102 (2015).

[21] Hughes EK, Gullone E, Dudley A, Tonge B (2010). A case-control study of emotion regulation and school refusal in children and adolescents. J. Early Adolesc..

[22] McShane G, Walter G, Rey JM (2001). Characteristics of adolescents with school refusal. Aust. N. Z. J. Psychiatry.

[23] Zhang C, Xue X, Zhao Y, Zhang X, Li T (2019). An improved association rule mining-based method for revealing operational problems of building heating, ventilation and air conditioning (HVAC) systems. Appl. Energy.

[24] Chiclana F (2018). ARM–AMO: An efficient association rule mining algorithm based on animal migration optimization. Knowl. Based Syst..

[25] Vasoya A, Koli N (2016). Mining of association rules on large database using distributed and parallel computing. Procedia Comput. Sci..

[26] Panesar SS, D'Souza RN, Yeh F-C, Fernandez-Miranda JC (2019). Machine learning versus logistic regression methods for 2-year mortality prognostication in a small, heterogeneous glioma database. World Neurosurg. X.

[27] Zhu S (2019). Research on data mining of education technical ability training for physical education students based on Apriori algorithm. Clust. Comput..

[28] Mirmozaffari M, Alinezhad A, Gilanpour A (2017). Data mining Apriori algorithm for heart disease prediction. Int. J. Comput. Commun. Instrument. Eng..

[29] Kasih, J., Ayub, M. & Susanto, S. Predicting students’ final passing results using the Apriori algorithm (2013)

[30] Jha J, Ragha L (2013). Educational data mining using improved apriori algorithm. Int. J. Inf. Comput. Technol..

[31] Angeline DMD (2013). Association rule generation for student performance analysis using apriori algorithm. SIJ Trans. Comput. Sci. Eng. Appl. (CSEA).

[32] Jeeva SC, Rajsingh EB (2016). Intelligent phishing url detection using association rule mining. Hum. Centric Comput. Inf. Sci..

[33] Raj S, Ramesh D, Sreenu M, Sethi KK (2020). EAFIM: Efficient Apriori-based frequent itemset mining algorithm on Spark for big transactional data. Knowl. Inf. Syst..

[34] Lemon SC, Roy J, Clark MA, Friedmann PD, Rakowski W (2003). Classification and regression tree analysis in public health: methodological review and comparison with logistic regression. Ann. Behav. Med..

[35] Epstein S (2020). School absenteeism as a risk factor for self-harm and suicidal ideation in children and adolescents: A systematic review and meta-analysis. Eur. Child Adolesc. Psychiatry.

[36] Havik T, Bru E, Ertesvåg SK (2015). Assessing reasons for school non-attendance. Scand. J. Educ. Res..

[37] Mauro, C. F. & Machell, K. A. in *Pediatric Anxiety Disorders* 439–460 (Elsevier, 2019).

[38] Adams D (2022). Child and parental mental health as correlates of school non-attendance and school refusal in children on the autism spectrum. J. Autism Dev. Disord..

[39] Filippello P, Buzzai C, Costa S, Sorrenti L (2019). School refusal and absenteeism: Perception of teacher behaviors, psychological basic needs, and academic achievement. Front. Psychol..

[40] Agyekum S (2019). Teacher–student relationships: The impact on high school students. Online Submiss..

[41] Gubbels JS (2011). Interaction between physical environment, social environment, and child characteristics in determining physical activity at child care. Health Psychol..

[42] Asai K, Asai K (2023). Therapeutic assessment with brief therapy: A single case study of an elementary student's school refusal. Int. J. Brief Ther. Fam. Sci..

[43] Balfanz R, Byrnes V (2012). Chronic absenteeism: Summarizing what we know from nationally available data. Baltim. Johns Hopkins Univ Cent. Soc. Organ. Sch..

[44] Bacon VR, Kearney CA (2020). School climate and student-based contextual learning factors as predictors of school absenteeism severity at multiple levels via CHAID analysis. Child. Youth Serv. Rev..

[45] Duke NN (2020). Adolescent adversity, school attendance and academic achievement: School connection and the potential for mitigating risk. J. Sch. Health.

[46] Chiu C-J (2016). The attitudes, impact, and learning needs of older adults using apps on touchscreen mobile devices: Results from a pilot study. Comput. Hum. Behav..

[47] Ko C-H, Yen J-Y, Liu S-C, Huang C-F, Yen C-F (2009). The associations between aggressive behaviors and Internet addiction and online activities in adolescents. J. Adolesc. Health.

[48] Thorén ES, Öberg M, Wänström G, Andersson G, Lunner T (2013). Internet access and use in adults with hearing loss. J. Med. Internet Res..

[49] Kavuk M, Keser H, Teker N (2011). Reviewing unethical behaviors of primary education students’ internet usage. Procedia Soc. Behav. Sci..

[50] Hafekost J (2016). Methodology of young minds matter: The second Australian child and adolescent survey of mental health and wellbeing. Aust. N. Z. J. Psychiatry.

[51] Lawrence D (2016). Key findings from the second Australian child and adolescent survey of mental health and wellbeing. Aust. N. Z. J. Psychiatry.

[52] Nguyen M-H, Ho M-T, Nguyen Q-YT, Vuong Q-H (2019). A dataset of students’ mental health and help-seeking behaviors in a multicultural environment. Data.

[53] Borah A, Nath B (2018). Identifying risk factors for adverse diseases using dynamic rare association rule mining. Expert Syst. Appl..

[54] Liu, X., Zhao, Y. & Sun, M. An improved apriori algorithm based on an evolution-communication tissue-like P system with promoters and inhibitors. *Discrete Dyn. Nat. Soc.* **2017** (2017).

[55] Yuan, X. in *AIP Conference Proceedings* 080005 (AIP Publishing LLC).

[56] Prajapati DJ, Garg S, Chauhan N (2017). Interesting association rule mining with consistent and inconsistent rule detection from big sales data in distributed environment. Future Comput. Inf. J..

